# Predicting cardiovascular toxicity in anti-PD-1/PD-L1 therapy: a risk factor analysis and model development

**DOI:** 10.3389/fcvm.2026.1731591

**Published:** 2026-03-03

**Authors:** Zhihui Yan, Juan Wang, Jianxiu Sun, Run Zhang, Jia Liu, Lihua Cao, Ming Zhang, Jiangtao Yu, Helei Hou, Wenzhong Zhang

**Affiliations:** 1 Department of Cardiology, The Affiliated Hospital of Qingdao University, Qingdao, Shandong, China; 2 Department of Cardiology, Linzi District Maternal and Child Health Care Hospital (QiDu Hospital), Zibo, Shandong, China; 3 Department of Cardiology, Qingdao Eighth People’s Hospital, Qingdao, Shandong, China; 4 Clinic for General Internal Medicine and Cardiology, Catholic Medical Center Koblenz-Montabaur, Koblenz, Germany; 5 Department of Oncology, The Affiliated Hospital of Qingdao University, Qingdao, Shandong, China

**Keywords:** cardiovascular toxicity, immune examination point inhibitors (ICIs), nomogram, risk factors, programmatic cell death protein-1 (PD-1), programming death ligand-1 (PD-L1)

## Abstract

**Purpose:**

This study aimed to investigate risk factors for cardiovascular toxicity following anti-PD-1/PD-L1 therapy and develop a predictive model.

**Methods:**

We retrospectively collected data from 2,665 patients with solid tumors treated with anti-PD-1/PD-L1 therapy at two-center between October 2018 and October 2023.We performed univariate and multivariate logistic regression to identify predictors of cardiovascular toxicity and developed a nomogram. Internal evaluation and internal validation were performed using receiver operating characteristic (ROC), decision curve analysis (DCA), calibration curve (CC) for internal evaluation and internal validation.

**Results:**

Univariate logistic regression identified the Systemic Inflammatory Response Index (SIRI;OR 2.26, 95% CI 1.19–4.27, *p* = 0.012), Eastern Cooperative Oncology Group performance status (ECOG;OR 9.67, 95% CI 3.04–30.69, *p* < 0.001), hypertension (OR 3.50, 95% CI 1.78–6.88, *p* < 0.001), diabetes (OR 2.52, 95% CI 1.13–5.66, *p* = 0.025), tumor metastasis (OR 0.17, 95% CI 0.08–0.39, *p* < 0.001), tumor stage (OR 0.40, 95% CI 0.21–0.76, *p* = 0.006), and sex (male vs. female)(OR 0.43, 95% CI 0.19–0.96, *p* = 0.040) as significant predictors. Multivariate analysis confirmed ECOG (OR 9.81, 95% CI 2.73–35.25, *p* < 0.001) and tumor metastasis (OR 0.26, 95% CI 0.10–0.71, *p* = 0.008) as independent predictors. Seven variables (*p* < 0.05 in univariate analysis) were included in a nomogram, which showed good accuracy and discrimination (AUC 0.77, 95% CI 0.70–0.85).

**Conclusions:**

SIRI, ECOG, hypertension, diabetes, tumor metastasis, tumor stage, and sex were significant predictors of cardiovascular toxicity. ECOG was an independent risk factor, while tumor metastasis was an independent protective factor, after adjusting for other covariates. The nomogram showed good accuracy and discrimination, with clinical utility for predicting cardiovascular toxicity risk in patients receiving anti-PD-1/PD-L1 therapy.

## 1 Introduction

In recent years, immune check point inhibitors (ICIs), represented by anti-programmed cell death-1 (PD-1) and programmed death-ligand 1 (PD-L1) therapies, have been developing rapidly in the field of tumor treatment, and have become another emerging therapeutic area for tumors after surgery, chemotherapy, radiotherapy, targeted therapy and anti-angiogenic therapy (1). Although PD-1/PD-L1 inhibitors have achieved remarkable success in cancer therapy, they can also trigger a spectrum of potentially serious adverse events resulting from excessive activation of the immune system, collectively referred to as immune-related adverse events (irAEs) (1, 2). Among them, cardiovascular irAEs, also known as cardiovascular toxicity, including myocarditis, pericarditis and vasculitis, heart failure, and acute myocardial infarction, currently occur in approximately 1%–1.5% of patients, and, although infrequent, the mortality rate is as high as 50% for myocarditis, and about 21% and 6% for pericarditis and vasculitis, respectively (3, 4). The mechanisms underlying ICI-related cardiovascular toxicity are dichotomous, involving direct T-cell-driven myocardial/vascular inflammation and indirect promotion of atherothrombosis via chronic inflammation (4–6). This distinction is clinically critical, as the former necessitates immunosuppression, while the latter warrants aggressive cardiovascular risk modification (7).To date, studies have not reached conclusive evidence regarding risk factors or biomarkers for cardiovascular toxicity that are specific to different ICI agents, tumor types, or organ systems. Several prediction models have been proposed to identify patients at risk of immune-related adverse events (irAEs) during immune checkpoint inhibitor therapy, many of which incorporate inflammatory biomarkers and have demonstrated improved predictive performance. However, cardiovascular-specific prediction models remain scarce, and this gap provides a strong rationale for evaluating inflammatory indices such as the systemic inflammatory response index (SIRI) and related markers for predicting cardiovascular toxicity in this setting (8, 9). Therefore, in order to reduce the adverse effects of cardiovascular toxicity of anti-PD-1/PD-L1 therapy, this paper takes 2,665 solid tumor patients after receiving anti-PD-1/PD-L1 immunotherapy as the research object, and constructs its risk prediction model through strict inclusion and exclusion criteria, which can help healthcare workers to identify the risk factors of cardiovascular toxicity related to PD-1/PD-L1 inhibitors at an early stage, and take preventive action on tumor It is of great significance for the choice of treatment plan and early preventive measures. This will allow immune checkpoint inhibitor therapy to play a greater role in benefiting tumor patients by improving their quality of life and reducing the burden of medical costs.

## 2 Methods

### 2.1 Subjects

This study is a retrospective, two-center study. The case data of 2,665 patients with solid tumors after receiving anti-PD-1/PD-L1 immunotherapy at the Affiliated Hospital of Qingdao University and the Eighth People's Hospital of Qingdao from October 2018 to October 2023 were screened, 60 patients with anti-PD-1/PD-L1 treatment-related cardiovascular toxicity were screened, and patients who did not experience any immune-related adverse events during the same period were screened in a 1:2 ratio 120 cases were included in the control group. Controls were matched 1:2 to cases based on age (±5 years), sex, tumour type, and treatment line, in addition to being irAE-free during follow-up. A total of 2,665 patients receiving ICI therapy were screened. During follow-up, 314 patients were lost to follow-up and excluded. Among the remaining patients, 60 developed cardiovascular toxicity and were included as cases. From the 2,291 patients without cardiovascular toxicity, 120 controls were selected at a 1:2 ratio and matched to cases. This matching design was chosen to enhance statistical power and reduce potential bias, as the number of cases was relatively limited, and increasing the number of controls improves the efficiency of estimation without substantially increasing cost or workload. Age 31–92 years, median age 65 (57, 70) years. Inclusion criteria: 1. Age ≥18 years; 2. Solid malignant tumor diagnosed by pathology or cytology; 3. Received at least 1 treatment with PD-1/PD-L1 inhibitors; 4. Relatively complete medical records with 1 year of follow up. Exclusion criteria: 1. Uncertain primary tumor, unclear pathological results and clinical stage; 2. Combination of multiple primary malignant tumors; 3. Acute infection during anti-PD-1/PD-L1 treatment; 4. Immunodeficiency or combination of autoimmune diseases; 5. Elevated cardiac biomarkers or cardiovascular symptoms and signs after immunotherapy, Symptoms and signs attributed to other diagnosed diseases[a multidisciplinary review (cardiology and oncology) attributed those findings clearly to pre-existing or alternative diagnoses rather than to ICI therapy]. With 7 potential independent variables, a minimum sample size of 70 was required. A total of 180 patients were included in this study. In the overall study population (*n* = 180), the underlying cancer types included lung cancer (*n* = 103), gastrointestinal malignancies (*n* = 51), gynecologic cancers (*n* = 9), and melanoma (*n* = 17).This retrospective study was approved by the hospital's medical ethics committee (approval number: QYFYWZLL29401) and conducted in accordance with the Declaration of Helsinki. Since this was a retrospective study, the Investigational Review Board waived the requirement for written informed consent.

### 2.2 Research methods

#### 2.2.1 Research tools

Potential risk factors for cardiovascular toxicity were identified through a literature review and expert consultation. The following patient information was collected: (1) General demographic characteristics: including sex, age, body mass index (BMI) [= weight (kg)/height^2^ (m^2^)]; (2) Disease-related conditions: Eastern Cooperative Oncology Group (ECOG) physical status score, tumor stage, tumor metastasis (yes/no), tumor stage was defined according to the AJCC 8th edition TNM staging system, and metastasis was recorded as present if distant metastasis was radiologically or pathologically confirmed; (3) Past history: including smoking history, alcohol use,combined hypertension, coronary heart disease, diabetes mellitus; (4) Laboratory indexes: SIRI (Systemic Inflammatory Response Index) = Neutrophils * Monocytes/Lymphocytes, SIRI was chosen after preliminary screening of several inflammatory indices (NLR, PLR, MLR), based on its stronger association with the outcome in univariate analysis and its hypothesized relevance to the systemic inflammatory state in ICI-related toxicity; (5) Treatment: whether combined treatment,“combined treatment” primarily referred to ICI combined with chemotherapy or with anti-angiogenic targeted therapy. Patients receiving concurrent tyrosine kinase inhibitors (TKIs) were excluded due to their independent cardiovascular risk profile.

Cardiovascular toxicity outcomes included myocarditis, non-malignant pericardial disease, acute coronary syndrome, congestive heart failure, atrioventricular block, supraventricular and ventricular arrhythmias, stress cardiomyopathy (Takotsubo syndrome), vasculitis, and venous thromboembolism. The composite endpoint “cardiovascular toxicity” included all cardiovascular events occurring during ICI treatment, irrespective of whether they were adjudicated as immune-mediated. Definitions for each event were based on the 2022 ESC Cardio-Oncology Guidelines, titled “Cancer Therapy-Related Cardiovascular Toxicity Definitions” and independently reviewed and confirmed by at least one senior cardiologist and one senior oncologist (10–12).

#### 2.2.2 Statistical methods

SPSS 27.0 (IBM SPSS Inc., Armonk, NY, USA) and R version 4.3.0 statistical software were applied to process and analyze the data. Continuous variables were tested for normality using the Shapiro–Wilk test. Measures that did not fit the normal distribution were expressed as median (interquartile spacing) [M (Q1,Q3)], and comparisons between groups were made using the Mann–Whitney U test for two independent samples. Categorical variables were presented as counts (percentages) and compared using the chi-square test or Fisher's exact test. Restricted cubic spline plots were used to assess non-linear relationships. All seven variables with *p* < 0.05 in univariate analysis were used as input variables for constructing the cardiovascular toxicity risk prediction model. R version 4.3.0 was used to develop the nomogram for predicting cardiovascular toxicity. Model calibration was assessed using the Hosmer-Lemeshow test and calibration curves. The predictive performance was evaluated using the receiver operating characteristic (ROC) curve and decision curve analysis (DCA). The sample size was determined based on the number of available cardiovascular toxicity events (*n* = 60). While traditional rules of thumb suggest 10–15 events per predictor variable (EPV) for logistic regression, recent methodological studies indicate that with appropriate variable selection and regularization techniques, models with lower EPV can still provide useful exploratory insights when validated internally (13, 14). We preselected variables with strong clinical rationale and *p* < 0.05 in univariable analysis, resulting in 7 predictors in the final model (EPV ≈ 8.6). Internal validation was performed using bootstrapping (500 replicates) to mitigate overfitting, and model performance was assessed via ROC, calibration curves, and decision curve analysis. A *p*-value < 0.05 was considered statistically significant.

## 3 Results

### 3.1 Basic characteristics of the study population

A total of 180 patients were included in this study, of which 136 (75.56%) were male and 44 (24.44%) were female. The median age of the included patients was 65 (57,70) years old; there were 85 cases (47.22%) with a history of smoking, 120 patients who did not experience cardiovascular toxicity, and 60 patients who experienced cardiovascular toxicity. Among the 60 patients with cardiovascular toxicity, myocarditis was the most common event (*n* = 35), followed by heart failure (*n* = 13), arrhythmias (*n* = 8), and acute coronary syndrome (*n* = 4). Table 1 list the demographic data and baseline characteristics. The results of the analysis of variance of the influential factors included in the study showed that the differences in age, sex, SIRI, ECOG, tumor stage, combined hypertension, combined diabetes, and tumor metastasis between the non-cardiovascular toxicity group and cardiovascular toxicity group were statistically significant (all *p* < 0.05). See Table 1.

**Table 1:** Comparison of the clinical data of the two groups of patients.

Variables	Total (*n* = 180)	Non-cardiovascular toxicity (*n* = 120)	Cardiovascular toxicity (*n* = 60)	Z/*χ*^2^	*p*
Age (year) M (Q_1_, Q_3_)	65.00 (57.00,70.00)	64.00 (55.00, 69.00)	67.00 (62.50, 72.00)	Z = −3.19	0.001
BMI (kg/m^2^) M (Q_1_, Q_3_)	22.48 (20.36,24.91)	22.40 (20.03, 24.93)	23.09 (20.71, 24.78)	Z = −0.85	0.397
SIRI (×10^9^) M (Q_1_, Q_3_)	1.25 (0.74, 2.31)	1.10 (0.66, 1.93)	1.46 (0.94, 3.77)	Z = −3.17	0.002
sex, *n* (%)				*χ*^2^ = 4.35	0.037
Male	136 (75.56)	85 (70.83)	51 (85.00)		
Female	44 (24.44)	35 (29.17)	9 (15.00)		
ECOG, *n* (%)				*χ*^2^ = 19.89	<.001
≤1	161 (89.44)	116 (96.67)	45 (75.00)		
>1	19 (10.56)	4 (3.33)	15 (25.00)		
Smoking history *n* (%)				*χ*^2^ = 1.35	0.246
No	95 (52.78)	67 (55.83)	28 (46.67)		
Yes	85 (47.22)	53 (44.17)	32 (53.33)		
Alcohol use *n* (%)				*χ*^2^ = 1.85	0.174
No	123 (68.33)	86 (71.67)	37 (61.67)		
Yes	57 (31.67)	34 (28.33)	23 (38.33)		
Tumorn stage, *n* (%)				χ^2^ = 7.88	0.005
Ⅱ–Ⅲ	59 (32.78)	31 (25.83)	28 (46.67)		
IV	121 (67.22)	89 (74.17)	32 (53.33)		
HBP, *n* (%)				*χ*^2^ = 13.85	<.001
No	128 (71.11)	96 (80.00)	32 (53.33)		
Yes	52 (28.89)	24 (20.00)	28 (46.67)		
DM, *n* (%)				*χ*^2^ = 5.26	0.022
No	151 (83.89)	106 (88.33)	45 (75.00)		
Yes	29 (16.11)	14 (11.67)	15 (25.00)		
CHD, *n* (%)				*χ*^2^ = 3.56	0.059
No	161 (89.44)	111 (92.50)	50 (83.33)		
Yes	19 (10.56)	9 (7.50)	10 (16.67)		
Combined treatment, *n* (%)				*χ*^2^ = 0.00	1.000
No	15 (8.33)	10 (8.33)	5 (8.33)		
Yes	165 (91.67)	110 (91.67)	55 (91.67)		
Tumor metastasis, *n* (%)				*χ*^2^ = 20.20	<.001
No	33 (18.33)	11 (9.17)	22 (36.67)		
Yes	147 (81.67)	109 (90.83)	38 (63.33)		

Z, Mann–Whitney test; *χ*^2^, Chi-square test; M, median; Q_1_, 1st quartile; Q_3_, 3st quartile; BMI, body mass index; SIRI, systemic inflammatory response index; ECOG, eastern cooperative oncology group physical status score; HBP, combined hypertension; CHD, coronary heart disease; DM, diabetes mellitus.

### 3.2 Construct cardiovascular toxicity risk prediction model

**Figure 1:**
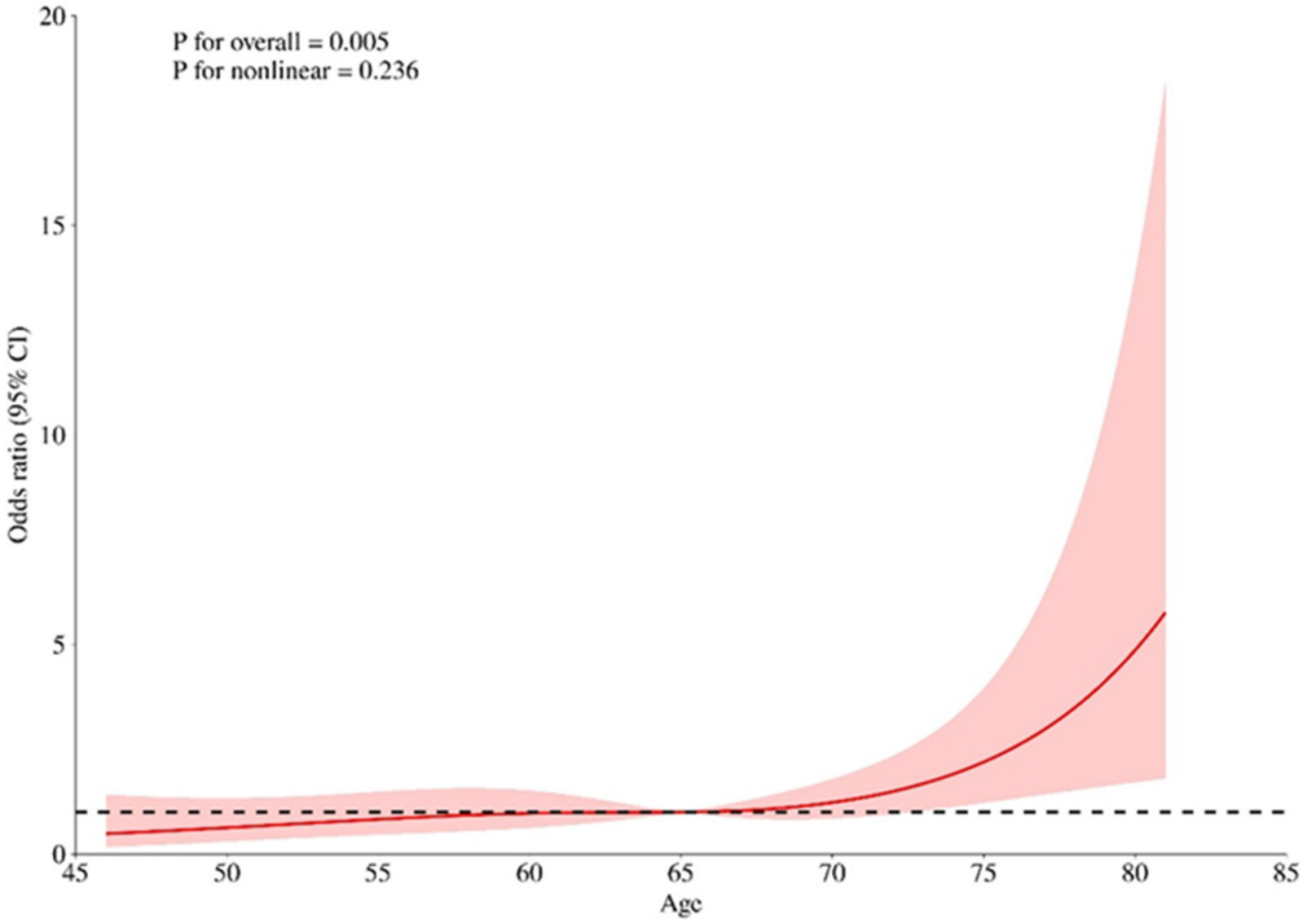
Non-linear relationship between age and cardiovascular toxicity outcome.

**Figure 2:**
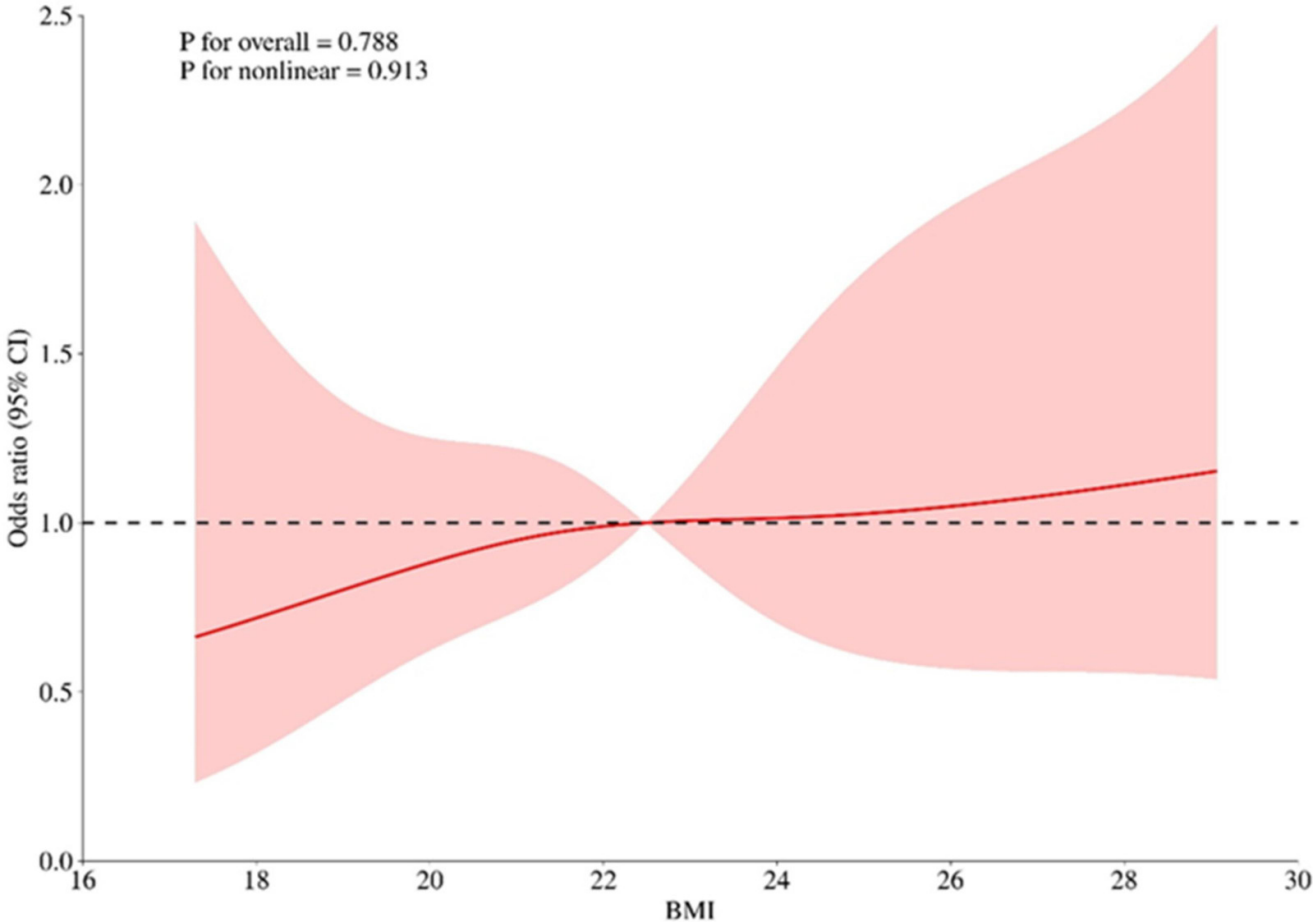
Non-linear relationship between BMI and cardiovascular toxicity outcome. BMI: body mass index.

**Figure 3:**
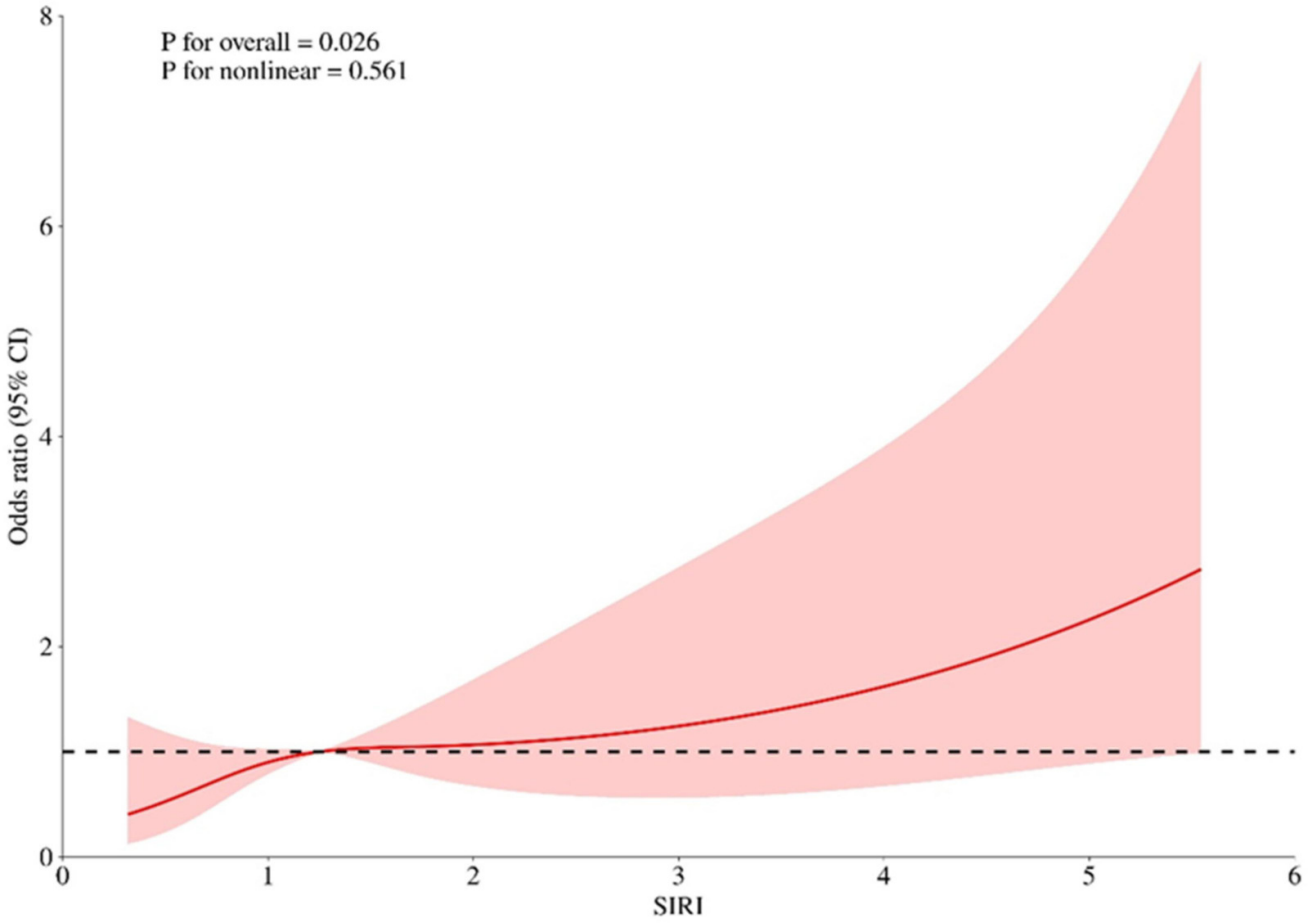
Non-linear relationship between SIRI and cardiovascular toxicity outcome. SIRI: systemic inflammatory response index.

**Figure 4:**
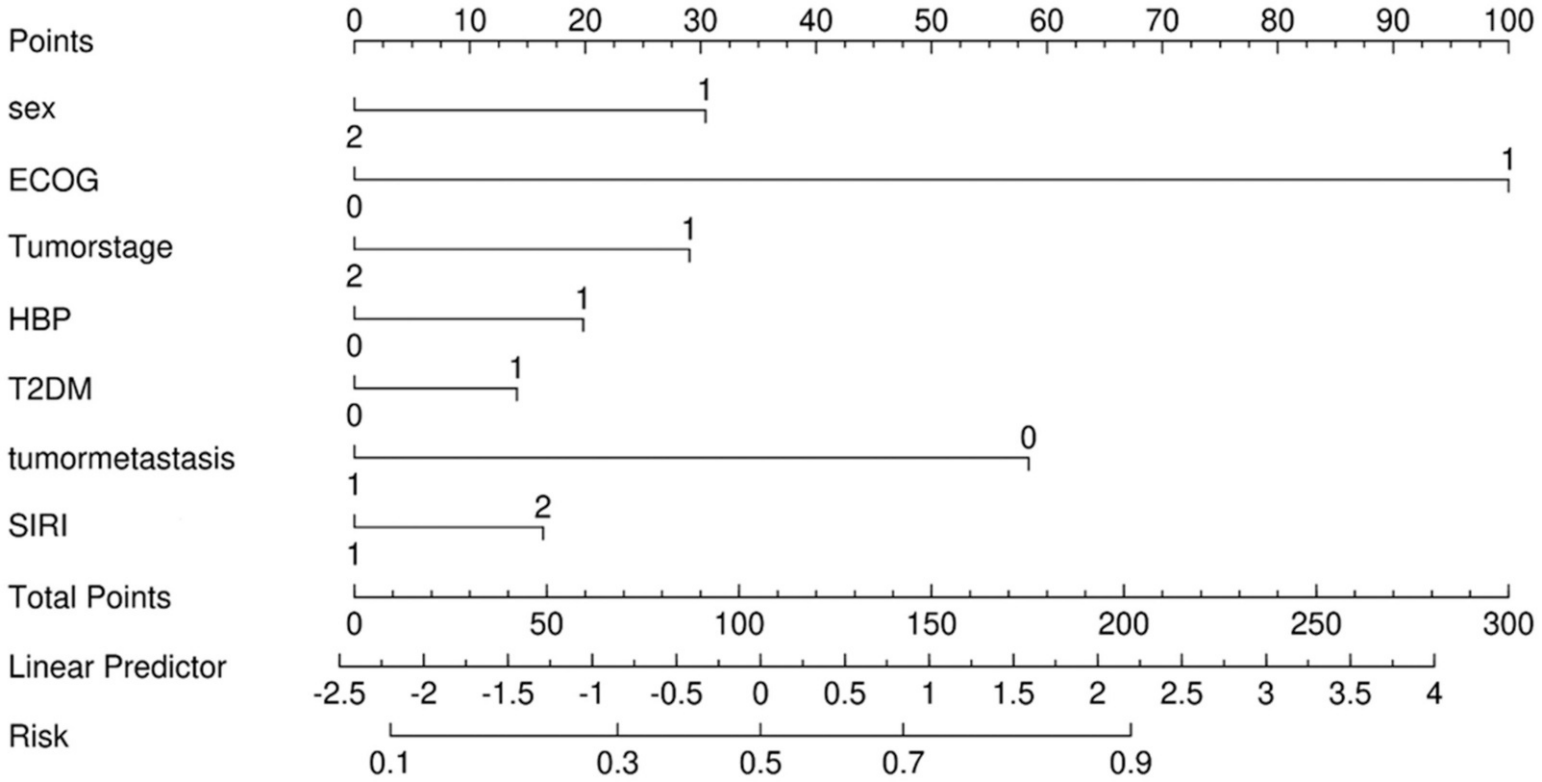
Nomogram prediction model for cardiovascular toxicity of anti-PD-1/PD-L1 therapy. Age (<65 years old assigned 1, ≥65 years old assigned 2), SIRI ( × 10^9^) (<1.25 assigned 1, ≥1.25 assigned 2, BMI (<22.48 kg/m_2_ assigned 1, ≥22.48 kg/m_2_ assigned 2) sex (female = 2, male = 1); ECOG (>1 assigned 1, ≤1 assigned 0), tumor stage (II–III assigned 1, IV assigned 2) combined with hypertension (no = 0, yes = 1); tumor metastasis (no = 0, yes = 1).

According to the relevant literature search and the recommendations of relevant experts this study intends to include the variables of one-factor logistic regression analysis *p* < 0.05 into the multifactorial logistic regression analysis, and among these variables, age, BMI, and SIRI are continuous numerical variables, and use the RCS (Restricted Cubic Spline) to evaluate whether the relationship between them and whether cardiovascular toxicity outcome occurs or not is nonlinear, and to determine its relationship with the Increased risk-related cutoff points, see Figures 1–3, age, BMI, SIRI p for nonlinear >0.05 (0.259, 0.836, 0.504), and therefore do not have a nonlinear relationship with cardiovascular toxicity outcomes. In order to construct the nomogram with high clinical utility, the cut-off values for age, BMI, and SIRI were determined based on clinical relevance, prior literature, and ROC curve analysis (Youden's index), relevant variables were converted into categorical formats with the following coding: age (<65 years old assigned 1, ≥65 years old assigned 2), SIRI (×10^9^) (<1.25 assigned 1, ≥1.25 assigned 2, and BMI (<22.48 kg/m^2^ assigned 1, ≥22.48 kg/m^2^ assigned 2) The other variables were assigned values: sex (female = 2, male = 1); ECOG (>1 assigned 1, ≤1 assigned 0), tumor stage (II-III assigned 1, Ⅳ assigned 2) combined with hypertension (no = 0, yes = 1); tumor metastasis (no = 0, yes = 1); These coded categories correspond directly to the variable axes (and their labels) shown in the nomogram (Figure 4). The re-assigned variables were subjected to single + multiple-factorial logistic regression analysis (the variables of single-factorial logistic regression analysis with *p* < 0.05 were included in the multiple-factorial logistic regression). factor logistic regression), and the results of the one-way logistic regression analysis showed that SIRI (OR = 2.26, 95% CI 1.19–4.27, *p* = 0.012), ECOG (OR = 9.67, 95% CI 3.04–30.69, *p* < 0.001), combined hypertension (OR = 3.50, 95% CI 1.78–6.88, *p* < 0.001), combined diabetes (OR = 2.52, 95% CI 1.13–5.66, *p* = 0.025), tumor metastasis (OR = 0.17, 95% CI 0.08–0.39, *p* < 0.001), tumor stage (OR = 0.40, 95% CI 0.21–0.76, *p* = 0.006), and sex (OR = 0.43, 95% CI 0.19–0.96, *p* = 0.40) were predictors of the occurrence of cardiovascular toxicity. The results of multifactorial logistic regression analysis showed that two indicators, ECOG (OR = 9.81, 95% CI 2.73–35.25, *p* < 0.001) and tumor metastasis (OR = 0.26, 95% CI 0.10–0.71, *p* = 0.008), were independent influences on cardiovascular toxicity, with ECOG as an independent cardiovascular toxicity risk factor and tumor metastasis was an independent protective factor for cardiovascular toxicity. See Table 2. Therefore, the seven variables of univariate logistic regression were used as input features to construct the cardiovascular toxicity risk prediction model by incorporating them into the nomogram.

**Table 2:** Results of univariate + multivariate logistic regression for risk of cardiovascular toxicity with anti-PD-1/PD-L1 therapy.

Variables	Univariate analysis					Multivariate analysis				
	β	S.E	Z	*P*	OR (95%CI)	β	S.E	Z	*P*	OR (95%CI)
Sex										
Male					1.00 (Reference)					1.00 (Reference)
Female	−0.85	0.41	−2.05	0.040	0.43 (0.19–0.96)	−0.69	0.51	−1.37	0.169	0.50 (0.19–1.34)
ECOG										
≤1					1.00 (Reference)					1.00 (Reference)
>1	2.27	0.59	3.85	<.001	9.67 (3.04–30.69)	2.28	0.65	3.50	<.001	9.81 (2.73–35.25)
Smoking history										
No					1.00 (Reference)					
Yes	0.37	0.32	1.16	0.246	1.44 (0.78–2.69)					
Alcohol use										
No					1.00 (Reference)					
Yes	0.45	0.33	1.36	0.175	1.57 (0.82–3.03)					
Tumor stage										
Ⅱ–Ⅲ					1.00 (Reference)					1.00 (Reference)
IV	−0.92	0.33	−2.77	0.006	0.40 (0.21–0.76)	−0.66	0.40	−1.64	0.100	0.52 (0.23–1.14)
HBP										
No					1.00 (Reference)					1.00 (Reference)
Yes	1.25	0.35	3.63	<.001	3.50 (1.78–6.88)	0.45	0.44	1.02	0.308	1.57 (0.66–3.75)
DM										
No					1.00 (Reference)					1.00 (Reference)
Yes	0.93	0.41	2.25	0.025	2.52 (1.13–5.66)	0.32	0.51	0.63	0.528	1.38 (0.51–3.75)
CHD										
No					1.00 (Reference)					
Yes	0.90	0.49	1.84	0.065	2.47 (0.94–6.44)					
Combined treatment										
No					1.00 (Reference)					
Yes	−0.00	0.57	−0.00	1.000	1.00 (0.33–3.07)					
Tumor metastasis										
No					1.00 (Reference)					1.00 (Reference)
Yes	−1.75	0.41	−4.21	<.001	0.17 (0.08–0.39)	−1.33	0.50	−2.65	0.008	0.26 (0.10–0.71)
Age (years)										
<65					1.00 (Reference)					
≥65	0.61	0.32	1.89	0.059	1.85 (0.98–3.49)					
SIRI										
<1.25					1.00 (Reference)					1.00 (Reference)
≥1.25	0.81	0.33	2.51	0.012	2.26 (1.19–4.27)	0.37	0.38	0.97	0.331	1.45 (0.68–3.08)
BMI										
<22.48 kg/m^2^					1.00 (Reference)					
≥22.48 kg/m^2^	0.30	0.32	0.95	0.343	1.35 (0.73–2.52)					

OR, odds ratio; CI, confidence interval; BMI, body mass index; SIRI, systemic inflammatory response index; ECOG, eastern cooperative oncology group physical status score; HBP, combined hypertension; CHD, coronary heart disease; DM, diabetes mellitus.

### 3.3 Cardiovascular toxicity prediction model constructed by nomogram and effect evaluation

**Figure 5:**
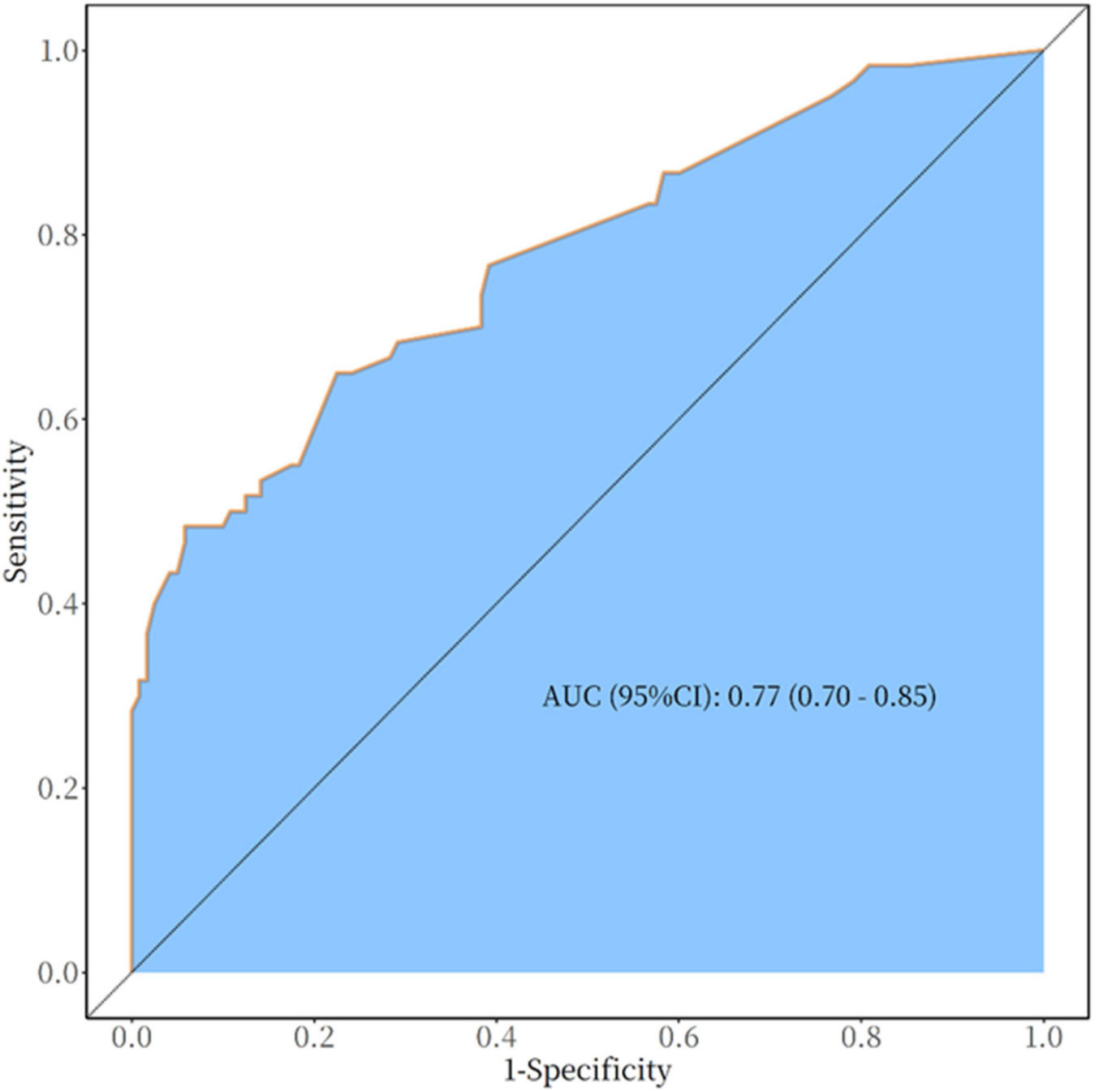
ROC curves of cardiovascular toxicity prediction model for anti-PD-1/PD-L1 therapy.

**Figure 6:**
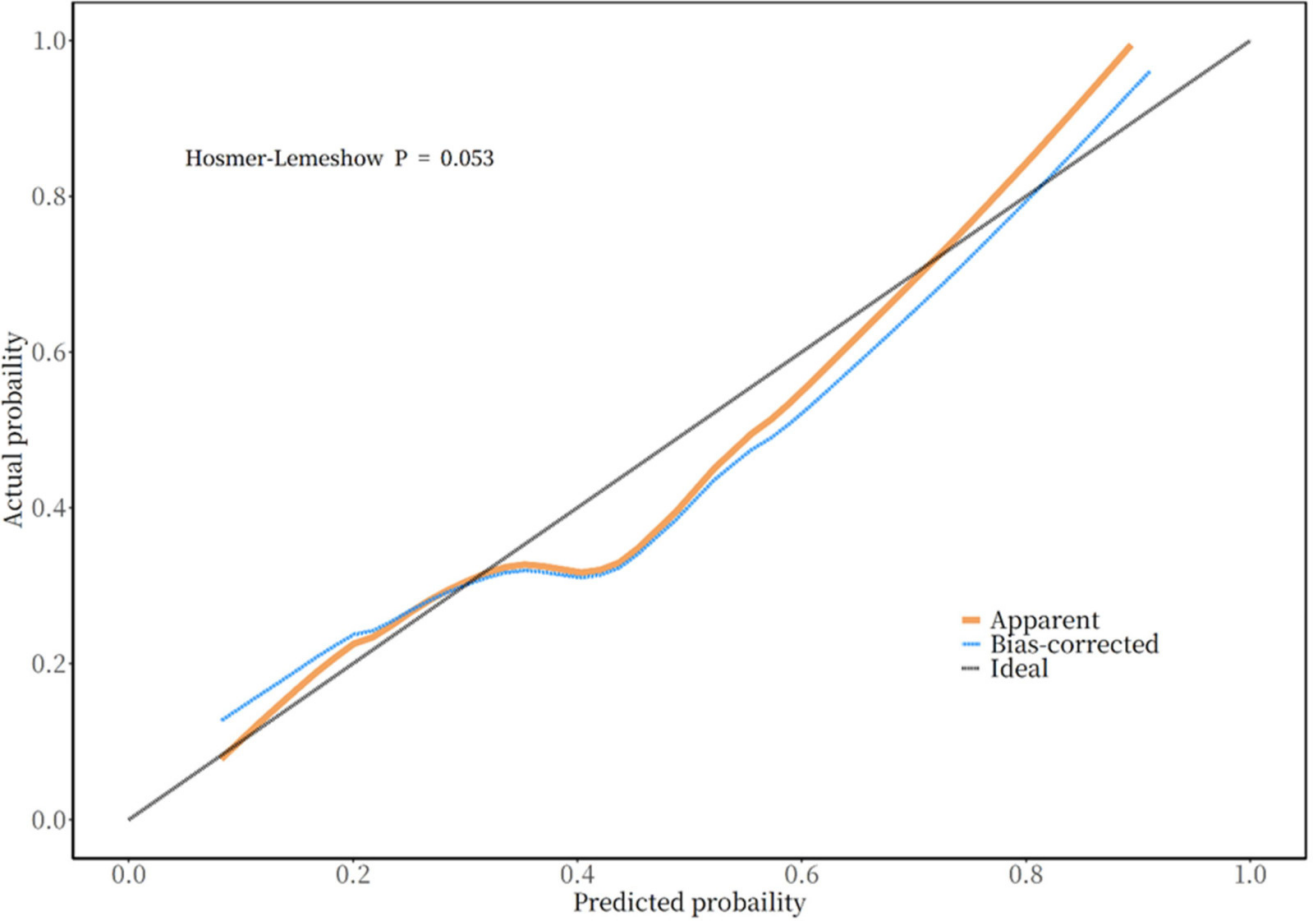
Calibration curve of the predictive model for cardiovascular toxicity of anti-PD-1/PD-L1 therapy.

**Figure 7:**
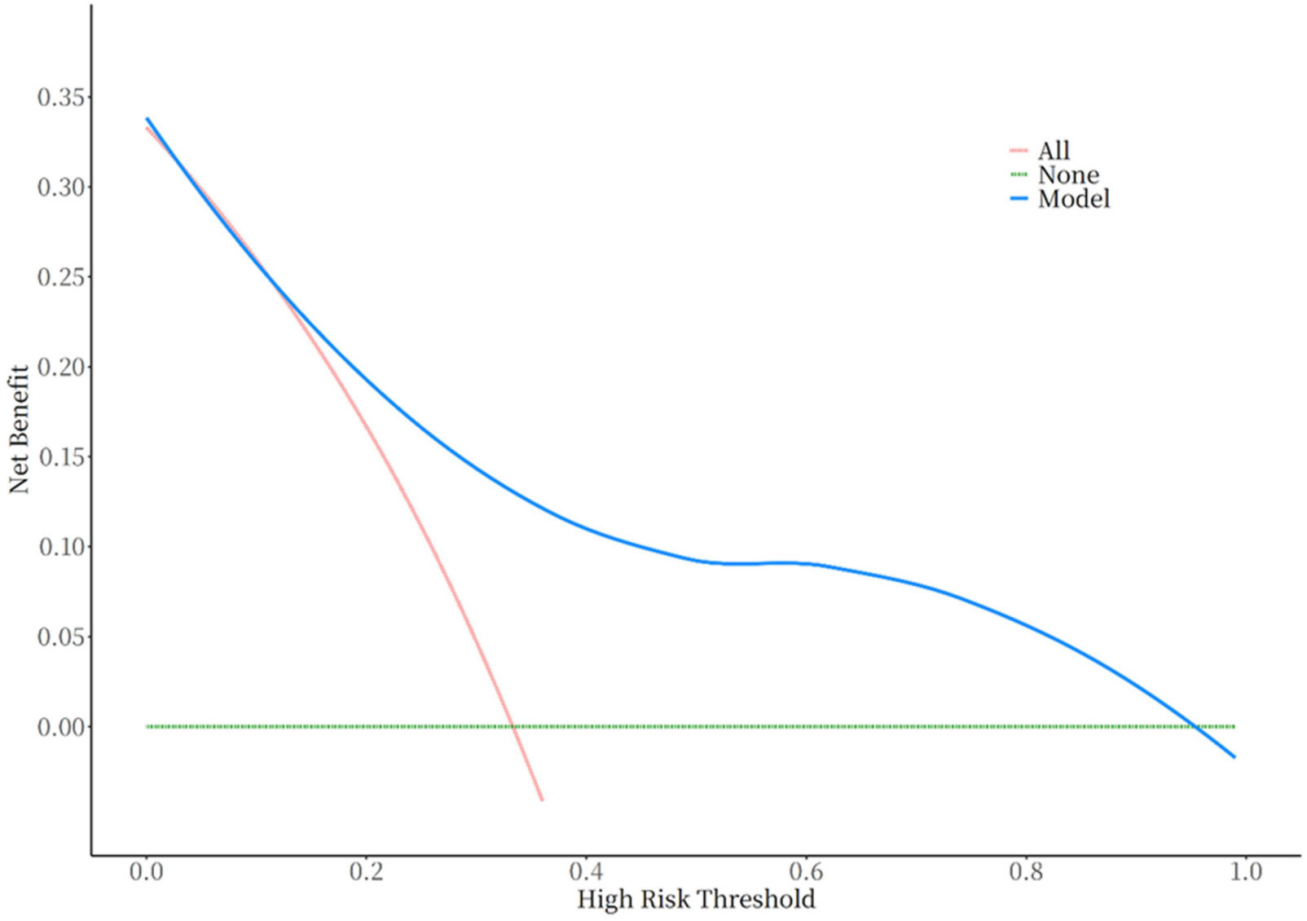
Decision making (DCA) curves for predictive modeling of cardiovascular toxicity of anti-PD-1/PD-L1 therapy.

The seven variables of one-way logistic regression were included in the nomogram to construct the cardiovascular toxicity risk prediction model, see Figure 4, with different values corresponding to different variables, and then the scores corresponding to each variable were summed up, and the resulting total score could be read on the total score value below, which corresponded to the probability associated with the occurrence of cardiovascular toxicity below. Thus, this model allows for individualized prediction for each patient. The cardiovascular toxicity prediction model constructed by the nomogram was validated, and the ROC curve was plotted, see Figure 5, with an AUC (Area Under Curve) of 0.77 and a 95% CI of 0.70–0.85, and its sensitivity, specificity, positive predictive value, negative predictive value and other indexes are shown in Table 3, and the predictive model was subjected to the Hosmer-Lemeshow test of the fitting effect, with a *χ*^2^ = 12.934 and a *p* = 0.053,the fit of the model is better (*p* > 0.05), which indicates that the accuracy and differentiation of the nomogram model is better, and the calibration curve of the internal evaluation and internal validation is better fit, revealing that there is a good agreement between the predictive probability of the model and the actual situation, showing the accuracy of the predictive model, see Figure 6.The DCA curve shows that this model has a better effect on the clinical benefit, see Figure 7.

**Table 3:** The cardiovascular toxicity prediction model AUC, accuracy, sensitivity, specificity, positive predictive value, negative predictive value and cut off.

AUC (95%CI)	Accuracy (95%CI)	Sensitivity (95%CI)	Specificity (95%CI)	PPV (95%CI)	NPV (95%CI)	Cut off
0.77 (0.70–0.85)	0.73 (0.66–0.80)	0.78 (0.70–0.85)	0.65 (0.53–0.77)	0.82 (0.74–0.89)	0.59 (0.47–0.71)	0.33

AUC, area under curve; PPV, positive predictive value; NPV, negative predictive value.

## 4 Discussion

The construction of cardiovascular toxicity risk prediction model.

Cardiovascular toxicity is a rare adverse reaction with a low incidence but high morbidity and mortality. Authoritative diagnostic criteria and clear mechanisms are still lacking, necessitating further research. Therefore, constructing a prediction model by systematically analyzing the risk factors of cardiovascular toxicity has high clinical value for the prevention and treatment of cardiovascular toxicity. These findings suggest that healthcare workers can use these risk factors to implement preventive and interventional measures. Early identification of cardiovascular toxicity risk can aid in treatment planning, improve patient outcomes, and reduce treatment costs.

Previous studies have shown that neutrophil to lymphocyte ratio (NLR) is an indicator of systemic inflammatory response. NLR is increased in patients with ICIs-induced myocarditis, and elevated NLR has been associated with major adverse cardiovascular events related to ICIs treatment (15–17). NLR reflects inflammatory activity. This is a possible mediator of severe myocarditis in cancer patients receiving ICIs. The systemic inflammatory response index (SIRI), integrating neutrophil, monocyte, and lymphocyte counts, has been widely recognized as a robust marker of systemic inflammation and immune status. Previous studies have demonstrated that SIRI independently predicts clinical outcomes in patients with advanced malignancies, including those receiving targeted therapies, underscoring its biological relevance as an inflammation-based prognostic indicator (18). In the context of immune checkpoint inhibitor therapy, such inflammation-driven immune dysregulation may also predispose patients to immune-related toxicities, providing a mechanistic rationale for evaluating SIRI in the prediction of cardiovascular toxicity. sIRI markers can be used to noninvasively assess the presence of pro-cancer inflammation in the tumor microenvironment and to predict the efficacy of targeted therapy and immunotherapy (19). Relevant studies have demonstrated that the development of irAE is associated with an association with baseline absolute lymphocyte count >2.6 k/uL [corrected (a)OR: 4.30], absolute monocyte count >0.29 k/uL (aOR: 2.34) and platelet count >145 k/uL (aOR: 2.23), neutrophil-to-lymphocyte ratio (NLR) ≤5.3 (aOR: 2.07) and monocyte-to-lymphocyte ratio (MLR) ≤0.73 (aOR: 2.96), as well as platelet-to-lymphocyte ratio ≤534 (aOR: 5.05) were associated (20).

### 4.1 ECOG

Higher ECOG scores were associated with worse 12-month overall survival (OS) and progression-free survival (PFS) in elderly patients with advanced cutaneous squamous cell carcinoma treated with ICIs in a previous real-world study (21). It is well known that ECOG physical status is an established prognostic marker for patients with advanced malignancies and correlates with OS (22).In the present study, we demonstrated that ECOG > 1 was an independent risk factor for cardiovascular toxicity ECOG (OR = 9.81, 95% CI 2.73–35.25, *p* < 0.001), which means that cardiovascular toxicity occurred 9.81 times more frequently in patients with ECOG > 1 than in patients with ECOG ≤ 1. This finding is clearly important when balancing the discussion of treatment benefits vs. risks or impact on quality of life. Therefore, patients with ECOG > 1 should fully consider the therapeutic benefit vs. the risk of cardiovascular toxicity when undergoing PD-1/PD-L1 therapy. This highlights the relevant role that ECOG still plays in treatment decisions.

### 4.2 Hypertension, diabetes mellitus

It has been shown that grade 1 and 2 hypertension without medication is associated with a higher risk of heart failure and other cardiovascular events in patients with breast, colorectal or gastric cancer (23, 24). Similarly, hypertension is a risk factor for chemotherapy-induced cardiomyopathy in oncology patients (25, 26). For patients with cardiovascular disease, hypertension and diabetes are a very important risk factor that promotes accelerated coronary atherosclerosis, plaque progression and rupture, myocardial remodeling, and myocardial ischemia and PD-1/PD-L1 has an important role in the above process. Atherosclerotic diseases are chronic inflammatory diseases, and PD-1/PD-L1-activated T-lymphocytes attack endothelial cells in addition to cardiomyocytes, promoting plaque progression and rupture as well as generating inflammation in the cardiac conduction system (15, 27). Therefore, PD-1/PD-L1 induces acute coronary syndrome and acute heart failure by damaging endothelial cells, accelerating plaque progression and rupture, and amplifying myocardial inflammation and myocardial ischemia (27–29). Our composite cardiovascular endpoint included myocarditis, arrhythmias, heart failure, acute coronary syndrome (ACS), venous thromboembolism, and others. While hypertension and diabetes are established risk factors for atherosclerotic events such as ACS, their association with other immune-related cardiotoxicities (e.g., myocarditis) may involve distinct pathways, such as systemic inflammation or endothelial dysfunction. Thus, the observed association likely reflects a heterogeneous risk profile across the composite outcome. The nomogram aims to capture shared clinical predictors that are readily available in practice, facilitating early risk stratification regardless of the specific cardiotoxicity subtype. Tumor Metastasis.

Unlike naïve T cells that circulate between secondary lymphoid tissues (i.e., lymph nodes) and blood, memory T cells circulate between lymphoid tissues, blood, and peripheral tissues (e.g., lungs, intestines, or skin) (30, 31). This allows memory T cells to respond directly and rapidly to the presence of pathogens in peripheral tissues. When tumors metastasize, memory *T* cells rapidly undergo activation and transfer to the corresponding tissues and organs to produce the appropriate anti-tumor effects. Recent studies have shown that CD4+ TCM cells (central memory CD4+ *T* cells) play a crucial role in cardioprotection during the treatment of ICIs (32), which is consistent with the results of the present study. In our analysis, tumor metastasis emerged as an independent protective factor against cardiovascular toxicity (OR = 0.26). While we hypothesize that memory *T*-cell redistribution and cardioprotective mechanisms may play a role, several non-causal explanations must be considered. These include selection bias (controls were irAE-free), competing risks such as shorter survival or earlier treatment discontinuation in metastatic patients, and potential confounding by tumor subtype, treatment regimen, or follow-up intensity. Therefore, this finding should be interpreted as hypothesis-generating rather than definitive. Future prospective studies with detailed time-to-event data, treatment duration adjustments, and larger sample sizes are needed to clarify whether metastasis truly confers biological protection or reflects methodological artifacts.

We observed a high risk of immune-related myocarditis, pericarditis, arrhythmias, coronary artery disease, and myocardial infarction in males in a retrospective study of immune checkpoint inhibitor-associated immune-associated adverse cardiac reactions, and we also found a sex-specific male pattern in adverse cardiac reports of arrhythmias, OR = 0.81 (95% CI 0.75–0.87, *p* < 0.001) (33, 34). The cardiovascular protective effects of estrogen have been confirmed by numerous studies both nationally and internationally (35, 36), which may predict that the protective effects of estrogen on the cardiovascular system may likewise be resistant to cardiovascular toxicity produced by immune checkpoint inhibitors.

### 4.3 Limitations

Several limitations of this study warrant consideration. First, the control group was selected from patients without immune-related adverse events (irAEs) during follow-up and matched to cases based on age, sex, tumor type, and treatment line. While this matched design improves comparability, it differs from a full cohort risk model and may influence the absolute risk estimates derived from the nomogram. Consequently, the model should be interpreted as a tool for relative risk stratification rather than for estimating absolute risk, with its clinical utility primarily focused on identifying high-risk individuals for intensified cardiovascular monitoring during anti-PD-1/PD-L1 therapy. Additionally, the retrospective nature of the study, the limited sample size, and the internal validation within a single cohort raise concerns regarding potential selection bias and overfitting. Therefore, external validation in prospective, independent, and larger populations is essential before the nomogram can be recommended for clinical application.

## 5 Conclusion

In this study, we analyzed risk factors for cardiovascular toxicity, including demographic, clinical, and inflammatory markers, and developed a nomogram prediction mode, which incorporating seven key factors (SIRI, ECOG, hypertension, diabetes, tumor metastasis, tumor stage, and sex), offers a user-friendly tool for clinicians to assess cardiovascular toxicity risk prior to anti-PD-1/PD-L1 therapy, potentially improving treatment strategies and patient outcomes.

## Data Availability

The raw data supporting the conclusions of this article will be made available by the authors, without undue reservation.
